# Beyond body mass index: a synthesis of lifestyle factors that may influence in vitro fertilisation outcomes

**DOI:** 10.12968/bjom.2023.31.8.436

**Published:** 2023-08-04

**Authors:** E Schneider, O Hamer, C Smith, J Hill

**Affiliations:** Liverpool University Hospitals NHS Foundation Trust; University of Central Lancashire

## Introduction

Body Mass Index (BMI) is used globally to determine access to fertility treatment including In Vitro Fertilization (IVF), with different countries imposing diverse BMI limits (1–3). Guidance in the United Kingdom imposes an IVF BMI threshold of 30 kg/m^2^, with those with obesity advised to lose weight in order to restore ovulation, increase their response to ovulation induction agents, and improve pregnancy outcomes (4–7). A recent systematic review suggests that women with a BMI greater than 30kg/m^2^ (classified as obese) have a clinical and statistically significant decreased probability of live birth following IVF, compared with women of healthy weight (BMI of 18.5–24.9 kg/m^2^) (8). That said, some researchers have questioned the scientific and ethical basis for the use of BMI thresholds in fertility treatment (2, 9), citing evidence that other factors may have a greater effect on the chance of success of IVF compared to BMI (2, 10).

Beyond BMI, a number of other lifestyle factors are thought to potentially impact on IVF outcomes, including smoking, alcohol consumption, caffeine, dietary patterns and exercise (11). Access criteria among most NHS policies for assisted conception stipulate the following lifestyle factors must be met in order to access NHS funded IVF (5–7):

- Female Body Mass Index between 19-30kg/m^2^ before IVF treatment can commence.
- Both partners must be non-smoking and not using any product containing nicotine in order to access any fertility treatment and must continue to be non-smoking throughout IVF treatment.
- Couples should give assurances that their alcohol intake is within Department of Health guidelines, and they are not using recreational drugs. Any evidence to the contrary will result in the termination of IVF treatment.

Although not included in UK IVF access criteria, guidance on excess caffeine consumption is included in some policy documents (5, 6). Within most criteria, factors such as physical activity, and dietary patterns are seldom mentioned, and only referred to for specific populations (e.g., women with hypothalamic pituitary failure, advising them to moderate their exercise levels if currently undertaking high levels of exercise) (5). This suggests that while some criteria may be evidence based, policy documents may not be considering the wide range of factors that could reduce the probability of live birth following IVF (10–14).

This article aims to briefly review the current evidence on how alcohol, recreational drugs, smoking, caffeine, dietary patterns, and physical activity may impact on IVF outcomes. In addition to a focus on BMI, it will consider whether a more holistic approach to assessing lifestyle may be warranted.

### The impact of alcohol consumption on IVF outcomes

The impact of alcohol consumption on IVF outcomes has been assessed in several recent studies (13, 15, 16). Consensus among these studies suggests that maternal consumption of alcohol is negatively associated with outcomes related to successful pregnancy following IVF treatment (13, 15). A recent systematic review found a possible association with high alcohol levels and decreased success rates in IVF treatment (13). The systematic review highlighted that when women consumed more than 84g of alcohol per week, their chance of achieving pregnancy after treatment was reduced (compared to abstainers) (13). Consistent with previous evidence, the findings also showed that maternal alcohol consumption was negatively (but not significantly) associated with live birth rates (13). Furthermore, paternal alcohol consumption may also be negatively associated with female partner's live birth after IVF (13). These findings concur with more recent research that highlights the importance of alcohol consumption as a key moderating factor for live birth following IVF (17). To summaries the current evidence base, data shows that reducing or abstaining from alcohol consumption prior to fertility treatment may improve IVF and pregnancy outcomes (13, 17). This is consistent with the recommendations of the Royal College of Obstetricians and Gynecologists, and the National Institute for Health and Care Excellence (NICE) who highlight the need to avoid drinking alcohol during pregnancy, particularly within the first twelve weeks (18, 19). The above evidence provides a rationale for NHS guidelines to consider alcohol as important moderating factor when deciding whether to provide IVF treatment.

### The impact of Illicit drug use on IVF outcomes

Over the past two decades there has been several studies that have evaluated the impact of illicit drug use on IVF and pregnancy outcomes (20–22). However, there are some limitations regarding the investigation of illicit drug use on reproductive function due to the ethical concerns of administration, and patient underreporting (23). That said, there is a consensus amongst literature that illicit drug use (e.g., marijuana, cocaine, heroin, methamphetamines etc.) during pregnancy should be avoided due to increased risk of adverse pregnancy outcomes (24–26).

Marijuana has been identified as a commonly used illicit substance during pregnancy (27). The use of marijuana during pregnancy is a concern given that active chemicals of the drug readily cross the placenta, impacting on the fetal brain (28). Furthermore, marijuana use can impact fertility, pregnancy, and fetal development by altering reproductive signaling and hormones (29). Exposure to the drug during pregnancy has been found to be associated with significantly lower birth weight, prematurity, small for gestational age, and congenital abnormalities compared non-users (30–32). Additionally, marijuana use during the first and second trimester has been linked to admission to neonatal ICU, and negative impacts on fetal and adolescent brain growth (33, 34). For women seeking IVF, marijuana is likely to reduce the chance of success given that regular use increases the risk of primary infertility when compared with non-users (35). In addition, studies show users of marijuana have significantly less embryo transfer and fewer oocytes retrieved which negatively impacts on the chances of IVF success (20).

Like marijuana, cocaine, heroin, and methamphetamine usage have also been associated with negative pregnancy and fertility outcomes, including infertility, placental abruption, low birth weight, preterm delivery, and neonatal mortality (24–26, 36–39). However, the extent of these associations is largely unknown because of a dearth of high-quality evidence (24). That said, some research suggests that the risk of infertility is substantially increased when women regularly use cocaine (from tubal abnormality) (34). Maternal cocaine abuse has also been associated with premature rupture of the female membrane, which can increase the risk of infant death (37, 38, 40). Similarly, methamphetamine abuse during pregnancy has been associated with premature rupture, preterm delivery and lower birth weight (41). Reductions in birth weight have also been associated with maternal heroin use (42). As a consequence of this evidence, there is a consensus amongst literature that advocates for a complete abstinence of these drugs prior to conception and during pregnancy (24, 37, 43).

With the likelihood that illicit drug use (e.g., marijuana, cocaine) negatively impacts on the success of IVF and increases the risk of primary infertility, there is a modest evidence base to consider them as an important moderating factor when making clinical decision relating to assisted conception. That said, further high-quality research is needed to strengthen the current evidence for the benefit of practice and policy.

### The impact of diet intake and eating behaviour on IVF outcomes

Dietary intake may impact IVF success rates by influencing various aspects of female fertility (11, 14). Studies have identified several potential pathways of effect, including changes in hormone levels, ovarian insufficiency, diminished ovarian reserve, and embryonic development (14). Evidence has suggested that two dietary factors of excess fat and dairy intake are associated with longer time to pregnancy and an increased risk of reproductive disorders (i.e., anovulatory infertility, endometriosis, and uterine leiomyomata) (14, 44, 45). Specifically, findings showed that trans fatty acid intake is associated with reduced conception rate (44, 46). Despite these findings, further research is needed to explore the associations between female dietary factors, infertility and pregnancy outcomes as recent findings have produced largely equivocal results (14).

Studies have shown that a Mediterranean diet, which is rich in vegetables, fruits, whole grains, fish, and unsaturated fats, may improve IVF outcomes for women under 35 years old who are not overweight or obese (47, 48). This diet may reduce inflammation, improve hormonal balance, and egg quality (47, 48). However, some studies have reported inconsistent or null associations between specific dietary patterns and IVF outcomes (e.g., Mediterranean diet inclusive of alcohol consumption) (49–51). Differences in study design, population characteristics, dietary assessment methods, IVF protocols, and outcome definitions may explain these discrepancies (50).

A 2022 systematic review by Muffone et al. examined 11 cohort studies and found a positive association between adherence to the Mediterranean diet and enhanced fertility outcomes (such as increased live births, pregnancy rates, and improved sperm concentration and count) (52). However, the strength of evidence was such that implications to practise recommendations to support the use of high adherence to the Mediterranean diet as a clinical intervention for improving fertility outcomes cannot be made. That said, a further systematic review by Kellow et al. (2022) suggested that adherence to the Mediterranean diet was linked to increases in live birth rate (53). Similarly, a review by Sanderman et al. (2022) found that higher adherence to the Mediterranean diet was linked to pregnancy and live birth following IVF treatment (14). With this evidence, there is a rationale to suggest that a Mediterranean diet (focus on reduced trans fatty acid intake) could be deemed an important factor for clinicians to consider when providing guidance on IVF treatment.

### The impact of caffeine on IVF outcomes

While the evidence on caffeine's impact on IVF fertility is inconsistent and limited, some studies have suggested that high caffeine intake may delay conception, reduce implantation rates, and alter ovarian function, endometrial receptivity, or embryo quality (50, 54). With that said, other studies have found no association or even a positive association between caffeine consumption and IVF success (55, 56). These discrepancies are likely to be due to differences in study design, population characteristics, caffeine sources, dose assessment, IVF protocols, and outcome definitions (51). As a consequence, further research is needed to establish causal links between caffeine intake and IVF fertility.

For women undergoing IVF, moderate caffeine intake from natural sources, such as coffee or tea, may be safe, while high caffeine consumption may increase the risk of infertility and reduce pregnancy outcomes (13). Recent evidence from a systematic review found no significant association between caffeine intake and IVF/ICSI outcomes (13). However, a possible threshold effect at 200 mg/day was observed for clinical pregnancy rate. Additional findings also suggested that caffeine intake from coffee or tea was not associated with IVF outcomes (13). Findings consistent with the review by Rao et al, found that moderate caffeine consumption (less than 300 mg/day) did not affect fecundity, fertility, pregnancy rate, or live birth rate (57). However, high consumption (more than 300 mg/day) reduced fecundity and pregnancy rate (57). From this evidence, there may be a rationale to suggest within NHS assisted conception policies that women undergoing IVF limit their caffeine intake to below 200mg per day (57). With that said, access criteria for NHS provided IVF may not yet include caffeine because of the limited strength and quality of evidence.

### The impact of physical activity on IVF outcomes

Exercise has been shown to have numerous positive effects on both physical and mental health (58–60). Exercise can help reduce stress levels (61, 62), increase life expectancy (63), prevent chronic conditions (64) and improve general health (65). In relation to pregnancy, adults receiving physical activity interventions may increase the likelihood of conception and live birth compared to usual care (66). A systematic review conducted recently corroborates these findings, indicating that physical activity can help lower the risk of infertility in healthy individuals (67). Moreover, when considering intensity, high- and moderate-intensity exercise may be more effective in reducing the risk of infertility than low-intensity exercise (67). The World Health Organization recommends that physical activity is continued during pregnancy and postpartum (68). Furthermore, that wherever possible during pregnancy sedentary time should be replaced with any intensity of physical activity (68).

The body of research into the effects of physical activity on IVF treatment has been growing steadily (69). There is a consensus amongst studies that suggests physically active women prior to undergoing IVF, have a higher chance of clinical pregnancy compared to their non-active counterparts (66, 69). Furthermore, exercise may also increase the chance of live birth (69). This is supported by more recent studies in this field, which have come to similar conclusions (70). However, the effects of physical activity during the treatment are less certain (70, 71). From this evidence, NHS assisted conception policies should consider the inclusion of physical activity given that it can increase the success rates of IVF and live birth.

### The impact of smoking on IVF outcomes

Smoking is a harmful habit that has serious consequences for the general health of individuals (72). It has been linked to a variety of diseases, such as cancer (73, 74) and heart disease (75, 76), and is a major risk factor for infertility (77, 78). In relation to pregnancy outcomes, smoking has been associated with an elevated probability of miscarriage, with an estimated relative risk increasing by 1% for every cigarette smoked daily (79). Even when exposed to second-hand smoke, the impact can result in a substantially increased risk of miscarriage which has an impact on guidance for couples (79). Furthermore, smoking has been linked to earlier onset of menopause and infertility (80, 81).

Studies have demonstrated that smoking may affect IVF treatment (82), as it has been shown to be associated with the impaired ovarian response, fertilization rate, implantation rates and the probability of achieving pregnancy (82). In more broader studies of the effect of smoking on assistive reproductive interventions, it has been shown to be associated with cycle cancellation with no embryo transfer, and cancellations before fresh oocyte retrieval or frozen embryo transfer (83), as well as affecting the mother’s potential to conceive (84). Smoking can also have a negative effect on a male’s sperm quality (85), where it can negatively affect sperm count, motility and morphology (85). Furthermore, modest evidence suggests that not only smoking habits during conception, but also historical habits, may negatively impact on the success of IVF (even when smoking cessation has occurred) (84).

Smoking during pregnancy can also have a negative effect on development (86), with smoking during pregnancy being shown to be associated with decreased second and third trimester head size, femur length, and estimated fetal weight (86). It can also lead to increased odds of cleft lip and/or palate (87), cardiovascular/heart defects, musculoskeletal defects, limb reduction defects, missing/extra digits, clubfoot, craniosynostosis, gastrointestinal defects, gastroschisis, anal atresia, hernia and undescended testes (88). Therefore, it is important to include smoking as a criteria for assisted conception access, as smoking history, current habits, and potential future habits could likely impact on the success of IVF.

## Discussion

In recent years, NHS services have placed a strong emphasis on criteria that mandates women to achieve a BMI below 30kg/m ^2^ in order to be eligible for IVF (5). This criterion includes non-smoking, BMI <30kg/m ^2^, alcohol intake within national guidelines and non-use of illicit drugs. However, these guidelines place little emphasis on factors such as dietary habits, activity levels and caffeine intake which arguably (together), may play an equal or greater role in improving the success of IVF and pregnancy outcomes (5–7). Although heightened BMI has been identified as a risk factor to fertility and newborn health, other factors such as alcohol consumption, dietary intake and physical activity may be equally as important to consider when trying to improve IVF outcomes (8, 11, 71, 84, 89, 90). The evidence presented in this commentary provides a rationale for NHS services (assisted conception) to adopt more robust criteria on dietary intake, alcohol consumption and physical activity to accompany existing criteria which focuses on BMI, smoking and illicit drug use. One method to achieve this could be the introduction of screening tools into assisted conception services which could support clinicians to identify patient dietary intake (including alcohol consumption) and physical activity levels (91, 92). The screening tool data could be used to review patient eligibility for IVF and identify any future intervention needs with the intension to improve IVF outcomes.

Over recent years, there have been several holistic approaches to improve IVF success (91, 93, 94). One protocol shown to be effective for adults undergoing assisted conception, is a holistic approach called FAST (Fertility ASsessment and advice Targeting lifestyle choices and behaviors) (95). The FAST approach employs approximately five questionnaires which screen for dietary intake, activity levels, psychological destress, general health and drug use (including alcohol consumption) (95). The approach assesses all factors and develops individual lifestyle practice interventions which often include motivational interviewing and telephone follow up to support healthy lifestyle change (increasing the success of IVF) (95). This approach is inclusive of weight management and weight reduction as a component, but would help to reduce weight stigma by shifting the focus towards a holistic approach to assisted conception (96). With an approach such as FAST, NHS trusts could begin to develop guidelines representing the wider range of factors which are of equal importance for improving IVF and pregnancy outcomes (95). New guidelines considering a holistic approach would move away from a narrow focus on a small number of factors (e.g., smoking, illicit drug use and BMI), towards an approach that reflects a more comprehensive scope of the current evidence.

Although several of the above lifestyle factors have been extensively explored, some require further investigation. This commentary highlights that the evidence related to caffeine and illicit drug use (as risk factors to fertility and IVF success) is limited because of a dearth of high-quality research. Consequently, further research is needed to strengthen associations between caffeine, illicit drug use and IVF outcomes (and pregnancy outcomes). Policy makers should be cautious to mandate criteria related to these factors, particularly caffeine intake (for patients seeking access to NHS provided IVF), given the level of uncertainty surrounding the quality and strength of evidence.

### Key Points

- High levels of alcohol consumption may decrease success rates in IVF treatment.
- There is no evidence of effect of caffeine intake (>200mg per day) on IVF outcomes.
- Physically active women undergoing IVF, have a higher chance of pregnancy and live birth compared to their non-active counterparts.
- Smoking is associated with fertilisation rate, and the reduced probability of achieving pregnancy.

### Reflective questions

1. What are the limitations and strengths of the evidence synthesised by this commentary article?
2. What are the limitations of a BMI of 30kg/m^2^ as an eligibility threshold for IVF treatment?
3. What other factors are important when developing guidance on assisted conception?
